# Islet Cell Biology, Regeneration, and Transplantation

**DOI:** 10.1155/2012/139787

**Published:** 2012-12-26

**Authors:** A. N. Balamurugan, Velayutham Kumaravel, Subbiah Pugazhenthi, Bashoo Naziruddin

**Affiliations:** ^1^Schulze Diabetes Institute, Department of Surgery, University of Minnesota, Minneapolis, MN 55455, USA; ^2^Alpha Hospital and Research Center, Institute of Diabetes and Endocrinology, Madurai, Tamilnadu 625009, India; ^3^Division of Endocrinology, Metabolism, and Diabetes, Department of Medicine, University of Colorado School of Medicine, Aurora, CO 80045, USA; ^4^Baylor Simmons Transplant Institute, Baylor University Medical Center, Dallas, TX 75246, USA

Research studies centered on the biology, regeneration, and transplantation of islets continue to shed significant understanding on the development of different forms of diabetes and provide further impetus for the quest to find a “cure.” Diabetes is a manifestation of an inadequate mass of insulin-producing pancreatic beta-cells. While type 1 is characterized by complete loss of beta-cells due to autoimmune attack on them, type 2 is characterized by a relative deficiency of beta-cells due to a decreased compensation for insulin resistance [1, 2]. Restoring beta-cell mass and reversing diabetes can be accomplished by two approaches: either by endogenous regeneration of beta-cells or transplantation of beta-cells from exogenous sources; recent advancements in science and technology have facilitated progress in both. In the first approach, while efforts to expand mature beta-cells *in vitro* have been met with limited success, regeneration of beta-cells from embryonic and adult stem cells, or pancreatic progenitor cells, has shown promise [2]. Understanding the role of beta-cell-specific transcription factors in the transdifferentiation to beta-cell phenotype is critical to further progress. Pharmacological approaches, employing growth factors, hormones, and small molecules, have also been shown to boost beta-cell proliferation and function. 

In the second approach, transplantation of isolated islets from cadaveric donor pancreas has proved to be an immediate and effective method for replacing depleted beta-cells in type 1 diabetic patients, allowing them to achieve independence from exogenous insulin administration [3, 4]. To preserve the transplanted beta-cell mass, however, islet transplant recipients require immunosuppression, which, under current regimens, are known to be beta-cell toxic. This limitation has ultimately led to poor long-term function of the transplanted islets and a disheartened medical community which is committed to providing a durable cure for patients. 

In this special issue, substantial developments made in different research areas aimed at overcoming current limitations of islet regeneration and transplantation are presented. Of the numerous papers received from this open submission format, selected papers have been recommended for publication after peer reviews. This special edition presents a collection of exciting papers that describe strategies to improve availability of beta-cells and islets for transplantation, and also to improve their posttransplant survival. 

It is clear that one of the major hurdles challenging further success in islet transplantation is the lack of suitable donor pancreases. This issue is compounded by poor long-term survival of allotransplanted islets. The review article by F.-C. Chou et al. summarizes many strategies developed to modulate immune response to transplanted islets. Gene therapy offers a powerful tool to engineer islet grafts to become resistant to apoptosis induced by inflammation and produce immunosuppressive molecules to attenuate T-cell response. In addition, the potential to develop patient-specific, autologous beta-cell replacement therapy by using iPSC-derived pancreatic beta-like cells is discussed. Key issues in this field which are presented in this paper include (i) duration and expression levels of targeted genes in islets, (ii) use of viral vectors for direct gene therapy that could lead to insertional mutagenesis and host immunogenicity, and (iii) poor efficiency of differentiation of insulin-secreting cells from stem cells.

Other recent studies have shown that long-term function of allogeneic islet transplants could be improved by effective induction immunosuppression and control of inflammation [4]. Further improvement of long-term success will require control of autologous and allogeneic immune response against islet grafts. Induction of donor-specific tolerance is a “holy grail” pursued by transplant immunologists to improve survival of both solid organ and cell transplants. S. Bhatt et al. have presented a comprehensive review of the attempts to induce donor-specific tolerance. Since the current immunosuppressive regimen used in islet transplantation could be toxic to beta-cells, the future of islet transplantation is dependent on the development of tolerance-inducing therapies. A tolerizing regimen that selectively targets donor-reactive T cells while expanding populations of regulatory T cells will result in better outcomes. Further investigation into inherently tolerogenic cells such as hepatic stellate cells, sertoli cells, and mesenchymal stem cells will aid in the design of therapies. 

Major causes of development of type 2 diabetes include excessive intake of food and lack of physical activity. Reduction in food intake which increases insulin sensitivity and improves glucose homeostasis is recommended to treat this metabolic disorder. The study by L. Belkacemi et al. investigated the effects of intermittent overnight fasting in streptozotocin-induced diabetic rats on glucose tolerance, plasma insulin, and homeostasis model assessment index. The study, which included an intermittent overnight fasting design (inspired by the daily fasting period during the Islamic Ramadan holiday), was recently reported to prevent the progressive deterioration of glucose tolerance otherwise taking place in sand rats exposed to a hypercaloric diet. The authors observed that the beta-cell mass, as well as individual beta-cell and islet area, was higher in intermittently fasting than in nonfasting STZ rats, and the percentage of apoptotic beta-cells was lower in the fasting STZ rats. Based on this study result, the authors proposed that intermittent fasting could represent a possible approach to prevent or minimize disturbances of glucose homeostasis in human subjects. 

The paper by S.-T. Chen et al. investigated the complementary role of hyperglycemia and p27kip1 suppression on islet beta-cell regeneration in a syngeneic mouse model. Glucose has been postulated to regulate cyclin D2 in pancreatic islet beta-cells and play a dominant role in beta-cell compensation; however, it is not yet clear how glucose controls the cell cycle of islet beta-cells. It has been reported that the suppression of both cdk-inhibitors p27kip1 and p18INK4c, but not p27Kip1 alone, promotes endocrine tumor formation in rodents. In this study, they used shRNAs to silence p27Kip1 and used hyperglycemia as a complementary factor to examine the synergistic effect of glucose and p27Kip1 on the adaptation of adult mice islets. They transduced adult islets with lentivirus-carrying shRNA to silence 80% of p27Kip1 protein, and the resultant suppression of p27Kip1 expression lasted for over 96 hours after infection. The study results suggested that adult mouse islet beta-cells can replicate when the metabolic demands increase, and there is a synergistic effect of hyperglycemia and concurrent suppression of p27Kip1 on islet beta-cell replication.

Beta-cell mass is maintained at optimal levels in the body through a slow turn-over rate. In humans, it has been shown that beta-cell mass expands several folds from birth and through the first three years of childhood, but thereafter this initial period, beta-cell replication potential declines markedly until adulthood. A critical barrier to progress in the treatment of diabetes is the lack of small-molecule drugs to induce beta-cell regeneration. Small molecule-induced beta-cell proliferation in humans could be an important way to achieve this goal; such compounds could be used to restore beta-cell mass *in vivo* or alternatively provide methods for *ex vivo* expansion of beta-cell numbers before transplantation. In the review article by A. Amedeo Vetere and B. Bridget K. Wagner, the authors present an overview of the current trends involving small-molecule approaches to induce beta-cell regeneration. For further understanding about the physiological proliferative behavior of human beta-cells, we can start to identify the molecular switches that could be used to foster the proliferation of beta-cells in humans.

In another interesting investigation into beta-cell mass equilibrium, the review article by E. Tarabra et al. describes the cellular counter-forces of beta-cell proliferation, neogenesis, and hypertrophy to increase beta-cell mass, while apoptosis and atrophy (reduced cell size) decrease beta-cell mass. They proposed that postnatal beta-cell mass responds to changing metabolic demands, carried out by an interaction of beta-cell replication (proliferation and/or neogenesis) and apoptosis, and this process is regulated by different growth factors/nutrients. Specifically, this review elaborated on principal hormones and nutrients, as well as downstream signaling pathways regulating beta-cell mass in the adult. They also reviewed the role of miRNAs in beta-cell mass regulation. The most studied miRNA in this contest was miR-375 overexpression, which was reported to attenuate proliferation of beta-cells and glucose-induced insulin secretion. In ob/ob mice in which miR-375 was deleted, a marked decrease in beta-cell mass resulted in severe insulin-deficient diabetes not found in ob/ob miR-375+ mice. Therefore, it is becoming clear that miR-375 targets a suit of genes that negatively regulate cell growth and proliferation and that aberrant loss of this miRNA leads to dramatic reduction of beta-cell mass.

Interestingly, generation of patient-specific beta-cells could also provide for a revolutionary type of treatment for patients with diabetes. The review article by X. Wang et al. focused on the significant applications of patient-specific therapy which include the engineering of new beta-cells from a patient's own cells, and thus, the elimination of the life-long usage of immunosuppressants, bioincompatibility, and disease transmission inherent with donor cells. Of course, transcription factors for pancreatic stem cell development and differentiation of beta-cells play a critical role in this process for they are essential for tailoring the transplantable beta-cells to function optimally. They concluded that the success of generating islet-like insulin-producing cell is largely achieved by building upon knowledge of the major steps in the differentiation of beta-cells during embryonic development of the pancreas. By applying multiple transcription factors, the available cells are coerced to differentiate into desired types in a unique delineation pathway, including across lineages, such as from fibroblasts into iPSs, or from one fully functional lineage to another, such as from fibroblasts into insulin-positive cells.

In conclusion, islet cell transplantation offers a potential cure for type 1 diabetes, but it is challenged by insufficient donor tissue and side effects of current immunosuppressive drugs [5, 6]. Therefore, alternative sources of insulin-producing cells and islet friendly immunosuppression are required to increase the efficiency and safety of this procedure. Beta-cells can be transdifferentiated from precursors or another heterologous (non-beta-cell) source to compensate for the reduced beta-cell function and insulin resistance experienced by diabetic patients. Fortunately, recent improvements in our understanding of islet cell biology and beta-cell regeneration have advanced the field of islet cell transplantation closer to our goal of finding a cure for diabetes [7]. 

*A. N. Balamurugan* *A. N. Balamurugan* *Velayutham Kumaravel* *Velayutham Kumaravel* *Subbiah Pugazhenthi* *Subbiah Pugazhenthi* *Bashoo Naziruddin* *Bashoo Naziruddin*
